# Case Report: Persistent drug-resistant *Pseudomonas aeruginosa* infection in a young post-kidney transplant patient that proved fatal

**DOI:** 10.3389/frtra.2025.1500066

**Published:** 2025-07-14

**Authors:** Supreeta R. Shettar, Mahadevaiah Neelambike Sumana, Manjunath S. Shetty, Yogeesh D. Maheshwarappa, Raghukanth G Reddy, Asha Srinivasan, Vamshi P Dharan, Gautam Kalyatanda, G. K. Megha

**Affiliations:** ^1^ JSS Medical College and Hospital, JSS Academy of Higher Education and Research, Mysuru, India; ^2^ Division of Nanoscience and Technology, School of Life Sciences, JSS Academy of Higher Education and Research, Mysuru, India; ^3^ Division of Infectious Disease & Global Medicine, University of Florida, Gainesville, FL, United States

**Keywords:** *Pseudomonas aeruginosa*, urinary tract infection, immunocompromised conditions, multidrug resistant organism (MDRO), recurrent hospitalization, recurrent UTIs

## Abstract

This case report highlights the management of recurrent urinary tract infections (UTIs) caused by multidrug-resistant (MDR) *Pseudomonas aeruginosa* in a post-renal transplant patient. Despite the challenges posed by antibiotic resistance, the patient was successfully treated with an extended infusion of meropenem, underscoring the efficacy of this approach in such difficult cases. The patient's recurrent infections required multiple hospitalizations and adjustments in treatment protocols, including the use of alternative antibiotics like fosfomycin and tailored immunosuppressive management to control both infection and rejection. This case is noteworthy for demonstrating the successful management of recurrent UTIs in the immunocompromised patient population, providing valuable insights into the treatment strategies that can be employed in similar clinical scenarios.

## Introduction

Infections are a significant issue in kidney transplant recipients (KTRs) (1). Over 80% of patients experience a bacterial infection within the first year after transplantation. Immunosuppressive therapy, which is essential to prevent acute and chronic rejection, increases the risk of infectious complications (2). In the first month post-transplant, these infections are often related to surgical complications and include wound infections, urinary tract infections (UTIs), pneumonia, and sepsis (3). The most common pathogens are multidrug-resistant (MDR) bacteria, especially Gram-negative rods (4). Late infections, typically occurring in the following five months, are caused by opportunistic bacteria, highlighting the serious consequences of immunosuppression. “UTIs are the most frequent infection after kidney transplantation and may lead to bloodstream infections”, which are reported to worsen graft function and reduce patient survival (5).

The incidence of UTIs after hospital discharge is over 90% in renal transplant recipients. The insertion of a double J catheter and Foley catheter are major risk factors for UTIs shortly after kidney transplantation (6). Additionally, an extended period of hemodialysis before transplantation and prolonged bladder catheterization increases the risk of UTIs (7). The timing and pattern of infections are influenced by the selection of immunosuppressive agents as well as the choice and duration of prophylactic antimicrobial therapy (8).

UTIs after kidney transplantation are typically caused by Gram-negative pathogens, with *Escherichia coli, Klebsiella pneumoniae, Proteus* species, and *Pseudomonas aeruginosa* being the most commonly isolated (9). UTIs can become more severe when caused by *P. aeruginosa*, especially MDR strains. *P. aeruginosa* is a significant uropathogen responsible for complicated UTIs, which can lead to fatal sepsis in older patients or immunocompromised individuals with deteriorating general conditions, such as those with diabetes or undergoing steroid or anticancer chemotherapy treatments.

*P. aeruginosa* is a common cause of severe healthcare-associated invasive infections, particularly pneumonia, bloodstream infections (BSIs), and complicated UTIs (cUTIs) (10). “The World Health Organization (WHO) has identified carbapenem-resistant *P. aeruginosa* (CRPA) as one of the priority pathogens for research and the development of new antibiotics” (11). Infections with *P. aeruginosa* that have limited treatment options are frequently reported in Intensive Care Units (ICUs) and long-term acute care hospitals, likely due to the extensive use of antimicrobials, which promotes the selection of this microorganism (12). “*P. aeruginosa* is among the top six pathogens responsible for deaths associated with antimicrobial resistance, alongside third-generation cephalosporin-resistant *E.coli, K.pneumoniae*, and Methicillin-resistant *Staphylococcus aureus* (MRSA)” (13, 14).

Several molecular mechanisms; intrinsic, acquired, and adaptive contribute to the antimicrobial resistance of *P. aeruginosa*. In a single clinical isolate, multiple resistance mechanisms can often coexist (10). Although each mechanism is associated with a specific class of antibiotics, these mechanisms collectively mediate varying levels of resistance across different antibiotic classes (10). Key contributors to the MDR phenotypes of *P. aeruginosa* isolates include a deficiency of outer membrane porins, overproduction of active efflux pumps, AmpC *β*-lactamase, extended-spectrum *β*-lactamases (ESBL), and Carbapenamase, particularly mettalo-*β*-lactamase (MBL) (15). Here, we describe an MDR *P. aeruginosa* isolated in renal transplant recipients with recurrent UTI treated with prolonged infusion of meropenem and intravenous fosfomycin. Written consent was obtained from the patient's family member to present this case.

## Case presentation

A 40-year-old male patient with a known history of hypertension, chronic kidney disease and autosomal dominant polycystic kidney disease (ADPKD) underwent live-related renal transplantation (donor-mother-in-law). A DJ stent was deployed during the surgery. Post-operatively, he was started on triple immunosuppressants (prednisolone + mycophenolate + tacrolimus). He had no post-operative complications. During the hospital stay, ultrasound sonography (USG) of the transplanted kidney was normal. His DJ stent and Foley catheter were removed on postoperative day 10 (refer to Table 1 for the treatment timeline). He was planned for discharge, but due to worsening serum creatinine levels, he was pulsed with one dose of methylprednisolone 250 mg. Following this, he developed a fever with chills and burning micturition. Blood, urine and drain fluid were sent for culture and sensitivity. Urine culture showed the growth of *Enterococci* (10^3^ CFU/ml). The antimicrobial susceptibility pattern of *Enterococci* is given in Table 2. Drain culture showed the growth of CRPA (refer to Table 3 for antimicrobial susceptibility results), and the blood culture showed no growth. He was initiated on injection meropenem 1 g TID (Three times a day) over a 3-h infusion for 7 days, following which his symptoms subsided. Repeat urine culture showed no growth. Repeat USG abdomen showed significant post-void residual urine in the urinary bladder, 200cc, with mild hydroureteronephrosis (HUN) in the transplant kidney, following which an alpha-blocker was added to the patient. At the time of discharge, repeat USG (Ultrasonography) showed a post-void residue of 25 ml with mild prominence of transplant renal pelvis with normal Doppler study. The patient was symptomatically better and was discharged with a serum creatinine of 1.68 mg/dl.

**Table 1:** Treatment timeline table.

Timeline	Case presentation	Treatment
It is a case of CKD and ADPKD and is a known case of hypotension		
	✓ Patient underwent live-related renal transplantation (donor-mother-in-law). ✓ A DJ stent was deployed during the surgery ✓ No post-operative complications ✓ USG of the transplanted kidney was normal	✓ Started on triple immunosuppressants Prednisolone + Mycophenolate + Tacrolimus
10th postoperative day	✓ DJ stent and Foley catheter were removed. ✓ Patient serum creatinine levels worsened ✓ Patient developed a fever with chills, and burning micturition ✓ Blood, urine and drain fluid were sent for culture and sensitivity	✓ He was pulsed with one dose of methylprednisolone 250mg
12th postoperative day	✓ Urine culture yielded the growth of *Enterococci* (10^3^CFU/ml) (Table 2) ✓ Drain yielded the growth of Carbapenem-Resistant *P. aeruginosa* ✓ Blood culture showed no growth ✓ Following the treatment symptoms subsided ✓ Repeat urine culture yielded no growth ✓ USG abdomen revealed significant post-void residual urine in urinary bladder 200cc with mild HUN in transplant kidney	✓ He was started on injection meropenem 1 g TID over a 3-h infusion for 7 days ✓ He was given with alpha-blocker
The patient was discharged with the post-void residue of 25 ml with mild prominence of transplant renal pelvis with normal Doppler study. The patient was symptomatically better and was discharged with a serum creatinine of 1.68 mg/dl		
Post-transplant day 35.	✓ He was presented to OPD with fever ✓ Urine culture showed the growth of MDR *P. aeruginosa* (Table 3)	✓ He was empirically treated with ciprofloxacin 500 mg BD for 5 days
Post-transplant day 41.	✓ Repeat urine culture showed the growth of MDR *P. aeruginosa* (Table 3)	✓ he was treated with meropenem 1 g TID over a 3-h infusion for 14 days
Following the treatment, his symptoms subsided and was later discharged		
2.5 months post-transplant	✓ Patient presented with complaints of persistent cough for 1 month ✓ Pulmonologist's opinion was taken and he was suspected of having drug (mycophenolate) -induced cough ✓ His serum creatinine levels are elevated and proteinuria is at 3.2 g/day ✓ He underwent a guided transplant kidney biopsy which showed features suggestive of rejection ✓ Urine culture yielded the growth of MDR *P. aeruginosa* (Table 3)	✓ Mycophenolate was changed to mycophenolate sodium ✓ He was treated with IV meropenem 1 g TID over a 3-h infusion for 7 days
The patient was symptomatically better and was later discharged		
3 months post-transplant	✓ He presented to OPD with complaints of pain in the abdomen, vomiting and fever for 3 days ✓ He was found to have drug-induced cytopenia ✓ Because of persistent vomiting, the gastroenterologist's opinion was sorted. ✓ He underwent an upper GI endoscopy, which showed a normal mucosal study ✓ Urine culture yielded MDR *P. aeruginosa* (Table 3) ✓ Creatinine levels were persistently elevated ✓ He underwent a transplant kidney biopsy: Borderline ACMR and ATN ✓ USG transplant kidney: Mild HUN with raised cortical echoes of the transplanted kidney ✓ Patient complained of a headache for which he underwent an MRI brain: meningioma	✓ Mycophenolate and Valganciclovir were stopped ✓ Patient was treated with meropenem 1 g TID over a 3-h infusion for 9 days ✓ He was pulsed with methylprednisolone for 3 days + oral steroids
A neurosurgeon's opinion was taken for the meningioma, and conservative management was advised. The patient was symptomatically better and was discharged.		
4-months post-transplant	✓ Patient presented to OPD with fever and chills for 3 days ✓ Urine culture yielded MDR *P. aeruginosa*	✓ He was treated with IV fosfomycin 4 g once daily for 7 days
The patient gradually improved and was later discharged		
4.5 months post-transplant	✓ Patient presented to OPD with fever and chills for 3 days ✓ Urine culture yielded MDR *P. aeruginosa* ✓ During his hospital stay he developed leucopenia ✓ Repeat urine culture yielded MDR *P. aeruginosa* (Table 3)	✓ He was treated with IV fosfomycin 4 g once daily for 7 days ✓ Mycophenolate was withheld ✓ He was injection meropenem 1 g TID over a 3-h infusion for 14 days
The patient was feeling symptomatically better and was later discharged		
5 months post-transplant	✓ Patient presented with OPD cough with minimal expectoration for the past 2- 3 days ✓ Vitals were stable ✓ Respiratory examination was normal ✓ Chest x-ray was normal ✓ During his hospital stay, he developed tachycardia, and tachypnea, along with a drop in blood oxygen saturation levels ✓ HR-CT revealed interstitial pneumonia ✓ BAL culture was negative ✓ Negative for CMV, PCP and TB ✓ Repeat HR-CT revealed features of diffuse ground glass opacities and extensive interstitial pneumonia ✓ COVID-19 and H1N1 RT-PCR was negative ✓ During his hospital stay, he had persistent pancytopenia and renal dysfunction ✓ Kidney biopsy showed severe ATN	✓ He was treated with injection meropenem 1 g TID over a 3-h infusion + oseltamivir 75 mg for 7 days + anti-tussive + anti-histamines + bronchodilators ✓ He was treated with cefepime/tazobactam 1.125 mg BD for 14 days + Tablet of antiflu for 10 days + Injection Fluconazole for 10 days + Tablet Doxycycline 100 mg BD for 5 days
The patient's total counts improved; he was started on mycophenolate and oral steroids. TAC level was 8.71 ng/ml, dose was reduced from 1.5 mg BD to 1.25 mg BD. The patient was symptomatically better and was later discharged.		
6 months post-transplant	✓ Patient presented to OPD with complaints of cough with sputum, fever and shortness of breath- grade III NYHA for 4–5 days ✓ He was found to have transplant kidney pyelonephritis with ADPKD cystic infection ✓ Urine and blood culture yielded the growth of MDR *P. aeruginosa* (Table 3) ✓ He had persistent fever spikes and worsening renal functions ✓ Transplant kidney biopsy, showed features suggestive of ABMR ✓ He developed metabolic acidosis ✓ Because of transplant kidney pyelonephritis-not responding to antibiotics ✓ He underwent cystoscopy + DJ stenting ✓ Patient developed sepsis with shock and severe metabolic acidosis ✓ He was shifted to isolation ICU and was started on inotropes and non-invasive ventilation (NIV) support	✓ He was treated with piperacillin + tazobactam 4.5 g stat dose followed by 2.25 g TID for 5 days ✓ Antibiotics escalated to injection of meropenem 2 g TID over a 3-h infusion ✓ He was treated with IV IgG for 5 days ✓ He was initiated on hemodialysis, during the hospital stay he underwent 7 sessions of hemodialysis ✓ Given their ongoing life-threatening infection, his immunosuppression was tapered and stopped
Given persistent tachypnea, the patient was intubated and was supported by mechanical ventilation. The patient's condition deteriorated and succumbed to illness		

CKD, chronic kidney disease; ADPKD, autosomal dominant polycystic kidney disease; USG, Ultrasonography; CFU, colony forming units; HUN, hydroureteronephrosis; OPD, outpatient department; MDR, multidrug-resistant; BD, bis in die/twice a day; TID, three times a day; IV, intravenous; ACMR, acute cellular mediated rejection; ATN, acute tubular necrosis; GI, gastro-intestinal; MRI, magnetic resonance imaging. HR-CT, high-resolution computed tomography; BAL, bronchoalveolar lavage; CMV, cytomegalo virus; PCP, *Pneumocystis* pneumonia; TB, tuberculosis; RT-PCR, real-time polymerase chain reaction; ABMR, antibody-mediated rejection; NYHA, New York Heart Association.

**Table 2:** Manual antimicrobial susceptibility pattern of *Enterococci*.

Antimicrobial agent	Interpretation
Penicillin	Resistant
Ampicillin	Resistant
Tetracycline	Resistant
Nitrofurantoin	Resistant
High-level Gentamicin	Resistant
Vancomycin	Sensitive
Teicoplanin	Sensitive
Linezolid	Sensitive

**Table 3:** Antimicrobial susceptibility pattern of *Pseudomonas aeruginosa .*

Antimicrobial agent	35th post-transplant day		41 days post-transplant		2.5, 3, 3.5, 4, 5 and 6-months post-transplant	
	MIC	Interpretation	MIC	Interpretation	MIC	Interpretation
Amikacin	≥64	Resistant	≥64	Resistant	≥64	Resistant
Cefepime	≥32	Resistant	≥32	Resistant	≥32	Resistant
Cefoparazone/sulbactam	≥64	Resistant	≥64	Resistant	≥64	Resistant
Ciprofloxacin	≥4	Resistant	≥4	Resistant	≥4	Resistant
Imipenem	≥16	Resistant	≥16	Resistant	≥16	Resistant
Meropenem	≥16	Resistant	≥16	Resistant	≥16	Resistant
Piperacillin/tazobactam	≥128	Resistant	≥128	Resistant	≥128	Resistant
Aztreonam	—	Resistant	—	Resistant	—	Resistant
Ceftazidime	≥64	Resistant	≥64	Resistant	≥64	Resistant
Levofloxacin	≥8	Resistant	≥8	Resistant	≥8	Resistant
Colistin	0.5	Intermediate	0.5	Intermediate	0.5	Intermediate
Fosfomycin	≥16	Resistant	≥16	Resistant	≥16	Resistant
Gentamicin	—	Intermediate	—	Resistant	—	Resistant

MIC, minimum inhibitory concentration.

On the 35th post-transplant day, the patient visited for follow-up on an outpatient department basis, urine culture showed the growth of MDR *P. aeruginosa* (refer to Table 3 for antimicrobial susceptibility results) for which he was empirically treated with ciprofloxacin 500 mg BD (Bis in Die/Twice a day) for 5 days. A repeat urine culture after one week still showed the growth of MDR *P. aeruginosa* (refer to Table 3 for antimicrobial susceptibility results), for which he was treated with meropenem 1 g TID over a 3-h infusion for 14 days, following which his symptoms subsided and he was later discharged.

At 2.5 months post-transplant, he presented with complaints of a persistent cough for 1 month. Because of lower respiratory tract infection (LRTI), a pulmonologist's opinion was taken, and it was suspected of having drug (mycophenolate) -induced cough, given that mycophenolate was changed to mycophenolate sodium. He had persistently elevated creatinine levels and proteinuria at 3.2 g/day. Because of this, he underwent a guided transplant kidney biopsy, which showed features suggestive of rejection. Given plenty of pus cells in the urine routine, a urine culture was sent, which showed the growth of MDR *P. aeruginosa* (refer to Table 3 for antimicrobial susceptibility results). He was treated with IV (Intravenous) antibiotic meropenem 1 g TID over a 3-h infusion for 7 days. The patient was symptomatically better and was later discharged.

At 3 months post-transplant, he presented with complaints of abdominal pain and fever for 3 days. On evaluation, he was found to have drug-induced cytopenia. Given this, tablet mofiran and tablet valren were withheld. Because of persistent vomiting, a gastroenterology opinion was taken, and he underwent an upper GI endoscopy, which showed a normal mucosal study. A urine culture was sent, which showed the growth of MDR *P. aeruginosa* (refer to Table 3 for antimicrobial susceptibility results), for which, after discussion with a consulting microbiologist, the patient was started on meropenem 1 g TID over a 3-h infusion for 9 days. The patient had persistent rising creatinine levels, for which he was pulsed with methylprednisolone for 3 days, and oral steroids were restarted. Later, he underwent a kidney transplant biopsy, which revealed borderline acute cellular-mediated rejection (ACMR) and acute tubular necrosis (ATN). USG transplant kidney showed features of mild HUN with raised cortical echoes of the transplanted kidney, for which a urologist's opinion was taken and advice was followed. The patient complained of a headache, for which a neurologist's opinion was taken, and the patient underwent a magnetic resonance imaging (MRI) of the brain with contrast, which revealed meningioma, for which the neurosurgeon advised conservative management. The patient was symptomatically better and was discharged.

At 4 months post-transplant, he presented with complaints of fever with chills for 3 days. Urine culture showed the growth of MDR *P. aeruginosa* (refer to Table 3 for antimicrobial susceptibility results), the patient was admitted and was started on IV antibiotic fosfomycin 4 g once daily for 7 days. The patient gradually improved and was later discharged.

At 4.5 months post-transplant, the patient presented with complaints of fever with chills for 3 days. On evaluation, he was found to have a UTI. The urine culture showed the growth of MDR *P. aeruginosa* (refer to Table 3 for antimicrobial susceptibility results), and the patient was treated with an injection of 4 g of fosfomycin once daily for 12 days. During the hospital stay patient developed leucopenia, for which mycophenolate mofityl was withheld. A repeat urine culture was sent, which still showed the growth of MDR *P. aeruginosa*, sensitive to meropenem. He was then treated with an injection of meropenem 1 g TID over a 3-h infusion for 14 days. The patient was feeling symptomatically better and was later discharged.

At 5 months post-transplant, he presented with complaints of cough with minimal expectoration for the past 2–3 days, whitish colour sputum-not blood stained, non-foul smelling, nasal stuffiness and common cold for 2 days with no other complaints. On initial evaluation, the patient's vitals were stable, the respiratory examination was normal, the chest x-ray was normal, and he was treated with injection meropenem 1 g TID over a 3-h infusion and oseltamivir 75 mg for 7 days with antitussive, antihistamines and bronchodilators. But the patient later started developing tachycardia, tachypnea, along with a drop in blood oxygen saturation levels (on room air-SPO_2_ level: 83%, with 4 L of 0_2_-SPO_2_ levels: 99%). High-resolution computed tomography (HRCT) chest was done, and was diagnosed with interstitial pneumonia. Bronchoscopy was done, and bronchoalveolar lavage (BAL) culture was sent, which was negative, even for cytomegalovirus syndrome (CMV), pneumocystis pneumonia (PCP) and tuberculosis (TB). Because of a persistent cough, a pulmonologist's opinion was taken, and repeat HRCT was done, which showed features of diffuse ground glass opacities and extensive interstitial pneumonia. Because of this, the sample was sent for COVID-19 and H1N1 RT-PCR, which were negative. During the hospital stay patient had persistent pancytopenia and renal dysfunction. The patient underwent a transplant kidney biopsy, which showed severe ATN. During the hospital stay, he was treated with IV antibiotics (injection of actamase 1.125 mg BD for 14 days, tablet antiflu for 10 days, injection of fluconazole for 10 days and tablet doxycycline 100 mg BD for 5 days). The patient's total counts improved; hence, he was started on mycophenolate mofitil and oral steroids. TAC level was 8.71 ng/ml, dose was reduced from 1.5 mg BD to 1.25 mg BD. The patient was symptomatically better and was later discharged.

At 6 months posttransplant, the patient presented with complaints of cough with sputum, fever and shortness of breath- grade III NYHA for 4–5 days. On evaluation, he was found to have transplant kidney pyelonephritis with ADPKD cystic infection, given which he was started on piperacillin + tazobactam 4.5 g stat dose followed by 2.25 g TID for 5 days. Later, because of urine and blood culture showing the growth of MDR *P. aeruginosa* (refer to Table 3 for antimicrobial susceptibility results), antibiotics were escalated to intravenous meropenem 2 g TID over a 3-h infusion. However patient had persistent fever spikes and worsening renal function, he underwent a transplant kidney biopsy, which showed features suggestive of antibody-mediated rejection (ABMR). The patient was treated with IV Ig G for 5 days [given ongoing urinary sepsis and cystic infection, plasma exchange (PLEX) was not possible]. However, the patient had worsening renal function and developed metabolic acidosis, hence, he was initiated on hemodialysis. During the hospital stay, he underwent 7 sessions of hemodialysis. Given the ongoing life-threatening infection, his immunosuppression was tapered and stopped (the risk of tapering immunosuppressants and graft dysfunction was explained to the attendees and the risk consent was taken and agreed to for the same). Because of a transplant kidney, pyelonephritis (not responding to IV antibiotics), a urology opinion was taken, and he underwent cystoscopy + DJ stenting. However, the patient developed sepsis with shock and severe metabolic acidosis, because of which he was shifted to the isolation ICU and was started on inotropes and non-invasive ventilation (NIV) support. Given persistent tachypnea, the patient was intubated and was supported by mechanical ventilation. The patient's condition deteriorated and succumbed to illness.

## Discussion

KTRs are predisposed to UTIs due to a combination of immunosuppression and urinary tract structural modifications such as urethral catheters, double-J stents, or ureteral anastomosis. Because of increased infection susceptibility and consequences associated with immunosuppressive medication, any symptomatic UTI in these patients, whether it affects the lower or upper urinary tract, is classified as complicated (7).

*P. aeruginosa* is especially alarming in patients with complicated UTIs because it can cause high rates of treatment failure and major consequences like relapse and antibiotic resistance (16). These individuals frequently carry MDR bacteria, which makes treatment challenging. *P. aeruginosa*, which has a high inherent resistance, is associated with poor prognoses and has spread globally, hampering treatment efforts due to a paucity of effective medicines (17).

According to multicenter cohort research by Gomila et al., younger individuals are more prone to have MDR *P. aeruginosa* than older patients. This was most likely because younger patients are more likely to receive strong antibiotic, surgical treatments and are more frequently admitted to intensive care units, which increases the chance of acquiring MDR strains. The study also discovered that procedures involving the urinary tract were a significant risk factor for MDR *P. aeruginosa*, and patients with this pathogen had a higher readmission rate (18).

The Centers for Disease Control and Prevention (CDC) reported 32,600 cases of MDR *P. aeruginosa* infections in hospitalized patients in the United States in 2017, with 2,700 deaths (12). The Infectious Disease Society of America (IDSA) 2023 guidelines recommend ceftolozane-tazobactam, ceftazidime-avibactam, imipenem-cilastatin-relebactum, and cefiderocol as preferable treatments for pyelonephritis and complicated UTIs caused by MDR *P. aeruginosa* (19). Similarly, the ICMR 2022 guidelines advocate combination therapy for severe infections caused by CRPA, which is only sensitive *in vitro* to polymyxins, aminoglycosides, or fosfomycin (20).

The most frequently indicated antibiotics for treating MDR Gram-negative bacteria are carbapenems (e.g., meropenem), colistin, fosfomycin, tigecycline, and aminoglycosides. Carbapenems, including imipenem and meropenem, are the preferred treatments for ESBL infections (20). Following intravenous infusion, carbapenems diffuse broadly into multiple bodily fluids, increasing their potency against a wide range of infections (21).

Zhanel et al. found that intravenous (IV) fosfomycin effectively treated both Gram-negative and Gram-positive infections. The most prevalent Gram-negative infections treated with IV fosfomycin were carbapenem-resistant Enterobacterials and MDR *P. aeruginosa*. Fosfomycin was primarily used as directed therapy because of resistance to first-line antimicrobial drugs, clinical failure of previous antimicrobial therapy, or side effects associated with previous antimicrobial medications. Despite its primary usage as a salvage treatment, IV fosfomycin was associated with rather good microbiological and clinical success rates (22).

Studies have suggested that fosfomycin has been associated with the treatment of MDR bacteria such as MDR *P. aeruginosa*, ESBL-producing *Enterobacteriaceae,* carbapenem-resistant *Klebsiella pneumoniae* (CRKP), vancomycin-resistant *Enterococcus faecium* (VRE), and MRSA. Another observational research by Zhanel et al. comprised 59 hospitalized patients who were given intravenous fosfomycin. Two patients were treated for complicated UTI caused by MDR *P. aeruginosa*, and both had clinical and microbiological cures (23).

“Due to the high level of intrinsic and acquired resistance among Pseudomonal isolates, higher doses or extended infusions of beta-lactams may be required to ensure early achievement of target concentrations and maximize the duration of drug concentration required to exceed the organism's minimum inhibitory concentration (MIC) in severe infections” (24). The IDSA and European Society of Clinical Microbiology and Infectious Diseases (ESCMID) guidelines propose greater doses for resistant and severe illnesses compared to susceptible and mild infections (24). A Monte Carlo simulation analysis concluded that complicated *P. aeruginosa* infections should be treated with a 2 g extended infusion of meropenem every 8 h rather than the conventional dose of 1 g every 8 h (25). Taccone et al. published a case study of a 70-year-old patient with septic shock caused by extensively drug-resistant *P. aeruginosa* who was treated with an extended infusion of meropenem (2 g every 8 h) and had favourable outcomes (26).

Zhao et al. conducted a study to assess the pharmacokinetics and pharmacodynamics of meropenem in critically ill patients and to determine if prolonged injection time improves meropenem therapy. “Prolonged infusion duration was advantageous when the MIC is ≤4 mg/L, but not for patients with drug-resistant or severe infections (MIC > 4 mg/L) who require a greater therapeutic goal. Their findings imply that prolonged injection duration may not always improve the success of antimicrobial therapy” (25).

Paul et al. conducted two randomized controlled trials (RCTs) comparing intravenous fosfomycin to piperacillin-tazobactam and meropenem. The piperacillin-tazobactam trial included patients with complicated UTIs or acute pyelonephritis, whereas the meropenem trial included patients with bacteremia. Both studies found no significant difference in clinical or microbiological cure rates between intravenous fosfomycin and the comparator drugs, meropenem and piperacillin-tazobactam (27).

Despite initial stability, the patient developed recurrent MDR infections, mycophenolate drug-induced adverse effects, and chronic renal impairment. Managing these issues necessitated several hospital stays, changes in the dosage of immunosuppressive medicine, and intensive antibiotic treatments. Unfortunately, despite multiple interventions, the patient's conditions deteriorated succumbing to illness (Lower respiratory tract infection, DTR-*Pseudomonas* infection of the urinary tract and graft rejection).

## Conclusion

This case study highlights the crucial need for close monitoring, early and appropriate antibiotic use in sufficient doses for sufficient duration and comprehensive management techniques to effectively combat reoccurring MDR infections in transplant patients. Though the standard guidelines for treatment for DTR-pseudomonas infection were followed, the patient succumbed due to multifactorial issues.

## Data Availability

The original contributions presented in the study are included in the article/Supplementary Material, further inquiries can be directed to the corresponding author.
